# Voxel-wise insights into early Alzheimer’s disease pathology progression: the association with APOE and memory decline

**DOI:** 10.1007/s11357-025-01610-z

**Published:** 2025-04-01

**Authors:** Maha Wybitul, Nicolas Langer, Christoph Hock, Anton Gietl, Valerie Treyer, for the Alzheimer’s Disease Neuroimaging Initiative, Michael Weiner, Paul Aisen, Ronald-Petersen, Clifford R. Jack, William Jagust, John Q. Trojanowski, Arthur W. Toga, Laurel Beckett, Robert C. Green, Andrew J. Saykin, John C. Morris, Leslie M. Shaw, Enchi Liu, Tom Montine, Ronald G. Thomas, Michael Donohue, Sarah Walter, Devon Gessert, Tamie Sather, Gus Jiminez, Danielle Harvey, Matthew Bernstein, Nick Fox, Paul Thompson, Norbert Schuff, Charles DeCarli, Bret Borowski, Jeff Gunter, Matt Senjem, Prashanthi Vemuri, David Jones, Kejal Kantarci, Chad Ward, Robert A. Koeppe, Norm Foster, Eric M. Reiman, Kewei Chen, Chet Mathis, Susan Landau, Nigel J. Cairns, Erin Householder, Lisa Taylor Reinwald, Virginia Lee, Magdalena Korecka, Michal Figurski, Karen Crawford, Scott Neu, Tatiana M. Foroud, Steven Potkin, Li Shen, Faber Kelley, Sungeun Kim, Kwangsik Nho, Zaven Kachaturian, Richard Frank, Peter J. Snyder, Susan Molchan, Jeffrey Kaye, Joseph Quinn, Betty Lind, Raina Carter, Sara Dolen, Lon S. Schneider, Sonia Pawluczyk, Mauricio Becerra, Liberty Teodoro, Bryan M. Spann, James Brewer, Helen Vanderswag, Adam Fleisher, Judith L. Heidebrink, Joanne L. Lord, Ronald Petersen, Sara S. Mason, Colleen S. Albers, David Knopman, Kris Johnson, Rachelle S. Doody, Javier Villanueva Meyer, Munir Chowdhury, Susan Rountree, Mimi Dang, Yaakov Stern, Lawrence S. Honig, Karen L. Bell, Beau Ances, Maria Carroll, Sue Leon, Erin Householder, Mark A. Mintun, Stacy Schneider, Angela Oliver, Randall Griffith, David Clark, David Geldmacher, John Brockington, Erik Roberson, Hillel Grossman, Effie Mitsis, Leyla deToledoMorrell, Raj C. Shah, Ranjan Duara, Daniel Varon, Maria T. Greig, Peggy Roberts, Marilyn Albert, Chiadi Onyike, Daniel D’Agostino, Stephanie Kielb, James E. Galvin, Dana M. Pogorele, Brittany Cerbone, Christina A. Michel, Henry Rusinek, Mony J. de Leon, Lidia Glodzik, Susan De Santi, P. Murali Doraiswamy, Jeffrey R. Petrella, Terence Z. Wong, Steven E. Arnold, Jason H. Karlawish, David A. Wolk, Charles D. Smith, Greg Jicha, Peter Hardy, Partha Sinha, Elizabeth Oates, Gary Conrad, Oscar L. Lopez, MaryAnn Oakley, Donna M. Simpson, Anton P. Porsteinsson, Bonnie S. Goldstein, Kim Martin, Kelly M. Makino, M. Saleem Ismail, Connie Brand, Ruth A. Mulnard, Gaby Thai, Catherine Mc Adams Ortiz, Kyle Womack, Dana Mathews, Mary Quiceno, Ramon Diaz Arrastia, Richard King, Myron Weiner, Kristen Martin Cook, Michael DeVous, Allan I. Levey, James J. Lah, Janet S. Cellar, Jeffrey M. Burns, Heather S. Anderson, Russell H. Swerdlow, Liana Apostolova, Kathleen Tingus, Ellen Woo, Daniel H. S. Silverman, Po H. Lu, George Bartzokis, Neill R. Graff Radford, Francine Parfitt, Tracy Kendall, Heather Johnson, Martin R. Farlow, Ann Marie Hake, Brandy R. Matthews, Scott Herring, Cynthia Hunt, Christopher H. van Dyck, Richard E. Carson, Martha G. MacAvoy, Howard Chertkow, Howard Bergman, Chris Hosein, Sandra Black, Bojana Stefanovic, Curtis Caldwell, Ging Yuek Robin Hsiung, Howard Feldman, Benita Mudge, Michele Assaly Past, Andrew Kertesz, John Rogers, Dick Trost, Charles Bernick, Donna Munic, Diana Kerwin, Marek Marsel Mesulam, Kristine Lipowski, Chuang Kuo Wu, Nancy Johnson, Carl Sadowsky, Walter Martinez, Teresa Villena, Raymond Scott Turner, Kathleen Johnson, Brigid Reynolds, Reisa A. Sperling, Keith A. Johnson, Gad Marshall, Meghan Frey, Jerome Yesavage, Joy L. Taylor, Barton Lane, Allyson Rosen, Jared Tinklenberg, Marwan N. Sabbagh, Christine M. Belden, Sandra A. Jacobson, Sherye A. Sirrel, Neil Kowall, Ronald Killiany, Andrew E. Budson, Alexander Norbash, Patricia Lynn Johnson, Thomas O. Obisesan, Saba Wolday, Joanne Allard, Alan Lerner, Paula Ogrocki, Leon Hudson, Evan Fletcher, Owen Carmichael, John Olichney, Charles DeCarli, Smita Kittur, Michael Borrie, T. Y. Lee, Rob Bartha, Sterling Johnson, Sanjay Asthana, Cynthia M. Carlsson, Steven G. Potkin, Adrian Preda, Dana Nguyen, Pierre Tariot, Adam Fleisher, Stephanie Reeder, Vernice Bates, Horacio Capote, Michelle Rainka, Douglas W. Scharre, Maria Kataki, Anahita Adeli, Earl A. Zimmerman, Dzintra Celmins, Alice D. Brown, Godfrey D. Pearlson, Karen Blank, Karen Anderson, Robert B. Santulli, Tamar J. Kitzmiller, Eben S. Schwartz, Kaycee M. Sink, Jeff D. Williamson, Pradeep Garg, Franklin Watkins, Brian R. Ott, Henry Querfurth, Geoffrey Tremont, Stephen Salloway, Paul Malloy, Stephen Correia, Howard J. Rosen, Bruce L. Miller, Jacobo Mintzer, Kenneth Spicer, David Bachman, Elizabether Finger, Stephen Pasternak, Irina Rachinsky, John Rogers, Andrew Kertesz, Dick Drost, Nunzio Pomara, Raymundo Hernando, Antero Sarrael, Susan K. Schultz, Laura L. Boles Ponto, Hyungsub Shim, Karen Elizabeth Smith, Norman Relkin, Gloria Chaing, Lisa Raudin, Amanda Smith, Kristin Fargher, Balebail Ashok Raj

**Affiliations:** 1 Institute for Regenerative Medicine, Faculty of Medicine, University of Zurich, 8952 Schlieren, Switzerland; 2 Department of Psychology, Faculty of Philosophy, University of Zurich, 8050 Zurich, Switzerland; 3 Methods of Plasticity Research, Department of Psychology, University of Zurich, 8050 Zurich, Switzerland; 4 Neurimmune, 8952 Schlieren, Switzerland; 5 University Hospital of Psychiatry Zurich, Geriatric Psychiatry and Psychotherapy, 8008 Zurich, Switzerland; 6 Department of Nuclear Medicine, University of Zurich, 8091 Zurich, Switzerland

**Keywords:** Alzheimer’s disease, Aging, Biomarker, Neuroimaging, Amyloid, Atrophy, APOE, Cognitive decline, Memory

## Abstract

Longitudinal investigation of the Apolipoprotein E (APOE) genotype’s impact on Alzheimer’s disease (AD) biomarker progression, focusing on amyloid beta (Aβ) accumulation and gray matter (GM) atrophy, integrating cognitive decline and baseline levels. Longitudinal florbetapir-PET and T1-weighted MRI data from 100 cognitively normal (CN) and mild cognitive impaired (MCI) participants both with considerable global Aβ accumulation (“high Aβ accumulators”) were analyzed using a voxel-wise approach. Associations of APOE genotype and memory decline with Aβ accumulation and GM atrophy were examined separately for each neuroimaging modality, controlling for baseline Aβ levels and diagnosis. Alternatively, the effect of baseline diagnosis, while controlling for memory decline, was investigated. A multimodal analysis evaluated interactions between genotype, memory decline, and GM atrophy on Aβ accumulation. High Aβ accumulators displayed extensive Aβ pathology predominantly in the medial orbito-frontal cortex, cingulate cortex, and precuneus, along with GM atrophy in temporal, occipital, orbito-frontal, and parietal areas. ɛ4 carriers with memory decline exhibited greater Aβ accumulation and GM atrophy in selective regions compared to non-carriers with memory decline, while no genotype difference was observed in individuals without decline. No interaction effect was observed for MCI diagnosis. Regional associations between the two biomarkers were similarly dependent on genotype and memory decline. ɛ4 carriers exhibiting memory decline present an accelerated neurobiological pattern at predementia stages, supporting early ɛ4 carrier monitoring and interventions in this at-risk group. Importantly, memory decline might be more informative than MCI regarding AD pathology progression emphasizing the importance of repeated cognitive assessments.

**Supplementary Information:**

The online version contains supplementary material available at 10.1007/s11357-025-01610-z.

## Introduction

Alzheimer’s disease (AD) is a progressive, neurodegenerative disorder and the most frequent dementia cause [1]. The disease manifests in a multitude of temporal and phenotypic disease courses [2, 3]. Its complexity has led to a growing body of research that attempted to map the spatiotemporal pattern of two of the major AD pathologies, amyloid beta (Aβ) and neurodegeneration represented by gray matter (GM) atrophy. With respect to Aβ’s temporal and spatial trajectories, the majority of past research agrees that the process of heightened Aβ burden typically begins in medio-frontal and cingulate areas, as well as in basal portions of the temporal cortex. Following, heightened Aβ burden spreads to the broader neocortex, with a late involvement of occipital regions. As the disease progresses, the distribution of Aβ extends to the striatal areas, the hippocampus, and entorhinal regions before finally impacting the remaining subcortex and cerebellum [4–7]. In comparison to Aβ pathology, the onset of GM atrophy has been repeatedly reported to occur at later stages of the disease course [8]. In mild cognitive impaired (MCI) and AD patients, GM loss has been described to occur in the temporal lobe, the precuneus, the parietal lobe, the occipital lobe, and the cerebellum based on cross-sectional and longitudinal studies [9–12]. A recent large cohort study, incorporating participants spanning the whole AD disease continuum, proposed additional GM loss in the thalamus/pulvinar and angular gyrus [13].

The spatial progression of these pathologies in relation to the Apolipoprotein E (APOE) genotype, the major genetic AD risk factor, is less well-characterized. A few studies investigated the influence of the APOE genotype on the spatial Aβ progression based on Aβ positron emission tomography (PET) imaging. Findings suggested that APOE ε4 carriers display higher and more extensive longitudinal Aβ increases, mainly within frontal, medio-temporal, and parietal regions, the cingulate cortex, and the precuneus based on voxel- and region-wise analyses [14–16]. However, these studies investigated relatively short time intervals and did not consider how baseline Aβ levels might modify subsequent progression (Jack et al., 2014; Landau et al., 2018; Lim & Mormino, 2017; Villain et al., 2012; Villemagne et al., 2013). Moreover, these investigations fail to distinguish between participants who effectively accumulate Aβ and those that do not; a statistical consideration that is increasingly recognized as critical when examining factors influencing Aβ accumulation [17, 18]. Previous research indicates that failing to account for this subgroup structure can lead to misleading conclusions about the progression of Aβ pathology [17–19]. An identification of “Aβ accumulators” might have additional clinical relevance. That is, this group has been recently discussed as being the optimal candidates for early-stage clinical trials [20, 21] given the associations between higher Aβ accumulation and more memory decline, increased tau pathology, and increased atrophy at preclinical stages [22–24]. Thus, Aβ accumulators might represent one of the earliest stages along the AD continuum offering a valuable window for potential intervention.

With respect to GM atrophy, there are several studies that have used T1-weighted magnetic resonance imaging (MRI) to examine APOE ε4’s effect on GM; however, the results are contradictory. Some investigations suggested more GM atrophy in ε4 carriers [25–28], while others show no effect [15, 16, 29]. Inconsistencies might arise from methodological differences, such as the use of cross-sectional data rather than longitudinal data and the inclusion or exclusion of MCI patients. Notably, many longitudinal investigations of both AD biomarkers neglect the aspect of cognitive decline over time. Most of the studies only account for the clinical diagnosis, despite existing evidence suggesting an impact of decreasing cognition on the association between these biomarkers and the APOE genotype [30–33]. Together, proper longitudinal examinations of how the APOE genotype influences the progression of the two biomarkers in actual Aβ accumulators representing an early disease stage would be highly valuable. Importantly, these investigations need to consider the additional aspects of baseline Aβ status and cognitive decline. Such research might enhance the knowledge about disease progression patterns in predementia stages, enhance the identification of regions of vulnerability, and facilitate the refinement of early intervention strategies.

The current study aimed at detecting the relation of the APOE genotype with the spatiotemporal progression pattern of Aβ pathology and of GM volume changes that reflect atrophy as measured by longitudinal [^18^F]florbetapir (FBP) PET imaging and longitudinal T1-weighted MRI, respectively. In comparison to previous investigations, we considered the interplay of memory decline and genotype while accounting for baseline Aβ levels. Data of cognitively normal (CN) and MCI participants were obtained from the Alzheimer’s Disease Neuroimaging Initiative (ADNI). The present study investigated participants termed as “high accumulators” that demonstrated considerable longitudinal increases in global Aβ values. Investigating this group might enhance our understanding about underlying pathological processes and their association with the major genetic AD risk factor as well as cognitive progression at a very early predementia stage. A voxel-wise analysis approach was applied to this subset that allowed for a comprehensive whole brain exploration without any a priori constraints. We primarily hypothesized a regional difference in Aβ accumulation and GM atrophy over time between APOE ε4 carriers and non-carriers. Importantly, we expected ε4 carriers to have higher regional Aβ accumulation rates and more atrophy than non-carriers dependent on whether they show signs of ongoing memory decline. Additionally, we examined how these associations compare to those influenced by the clinical diagnosis of MCI. Due to the study’s focus on longitudinal biomarker changes, we assumed that baseline clinical diagnosis might be less relevant than actual memory decline. Particularly, an MCI diagnosis is a single time-point classification and has been proven to be highly heterogeneous in its cognitive and pathological trajectories [34]. In contrast, memory decline is a dynamic measure and manifests before formal MCI criteria are met [23, 24, 35, 36]. Thus, focusing on longitudinal cognitive changes might align more closely with the temporal progression of AD biomarkers than relying solely on a baseline label [23, 24]. A better understanding of these complex inter-relations and how they influence the spatiotemporal patterns of Aβ, and GM atrophy would not only improve our understanding of the biomarker dynamics but also inform targeted AD intervention strategies.

## Materials and methods

### Study population

Data for this investigation were obtained from the Alzheimer’s Disease Neuroimaging Initiative which is a large longitudinal multi-center cohort study. The ADNI data repository encompasses data from various protocols (ADNI 1, 2, 3, ADNI Go). The ADNI was launched in 2003 as a public–private partnership, led by Principal Investigator Michael W. Weiner, MD. ADNI’s primary goal has been to assess whether serial MRI, PET, other biological markers, and clinical and neuropsychological assessment can be combined to measure the progression of MCI and early AD. Up-to-date information can be found at www.adni-info.org. The ADNI data are available to the scientific community without embargo at http://adni.loni.usc.edu/data-samples/access-data/ after approval by the Data Sharing and Publications Committee and adherence to the ADNI Data Use Agreement and publication policies. For the current analysis, we initially examined 543 participants who received an Aβ-PET at three or more time points. The further selection process primarily focused on identifying participants with considerable increases in Aβ accumulation over time. Thus, global Aβ levels were extracted in terms of centiloid (CL) values [37] and the individuals further included in the current investigation were those characterized as showing high increases in global Aβ levels over time. Specifically, we employed a *k*-means clustering algorithm designed for longitudinal data that uses the actual shape of the individual Aβ trajectories to categorize participants (R package ‘kmlShape’ version 0.9.5). The whole clustering approach is described in more detail in [19]. This approach resulted in a subsample of 100 individuals termed as “high Aβ accumulators.” The total mean annual CL increase rate of the resulting cohort was 6.45 (*SD* = 3.06). The remaining participants were classified as having no or only small increases in global CL values over time (*M* = − 0.11, *SD* = 3.14) and were therefore not included in the current study. We obtained MRI scans, Aβ-PET scans, APOE genotyping, demographics, and clinical information for all participants. Participants were characterized as either CN, or as already cognitively impaired with a diagnosis of mild cognitive impairment MCI (early or late subgroups) or subjective memory concern (SMC) at baseline. This classification was based on the criteria set by the ADNI consortium. In detail, participants were defined as CN based on a Mini Mental State Examination (MMSE) score between 24 and 30, a Clinical Dementia Rating (CDR) of 0; they had to be non-MCI and to have no objective memory loss based on the delayed recall of one paragraph from the Wechsler Memory Scale (WMS) Logical Memory II. SMC was defined based on MMSE scores between 24 and 30, a significant subjective memory concern reported by the participant, an informant, or a clinician, a Charlson Comorbidity Index score ≥ 16, a CDR of 0; participants had to be non-MCI and had to have no objective memory loss based on the delayed recall of one paragraph from the WMS Logical Memory II. MCI was defined by a memory complaint and MMSE scores between 24 and 30, participants had to have objective memory loss measured by the WMS Logical Memory II, a CDR of 0.5, but general cognition and activities of daily living had to be preserved. All participants had to be non-depressed and to have an absence of dementia. In the following, MCI and SMC patients will be referred to as MCI participants as SMC patients might represent an early disease stage [38] with heightened risk for developing objective cognitive impairment [39] and AD [e.g., 40]. A sensitivity analysis excluding the SMC participants was conducted for the major analyses to ensure the results’ robustness. At baseline, patients were aged between 55 and 90 years. Full information regarding the ADNI inclusion/exclusion criteria and recruitment can be accessed at http://adni.loni.usc.edu/. Institutional review boards granted ethical approval.

### APOE genotyping

A detailed description of APOE genotyping methods can be retrieved from https://adni.loni.usc.edu/data-samples/data-types/. According to the presence of an ɛ4 allele, participants with the ɛ2/ɛ3 or the ɛ3/ɛ3 genotypes were grouped together as the ɛ4 non-carriers, and participants with the ɛ3/ɛ4 or the ɛ4/ɛ4 genotypes were grouped together as the ɛ4 carriers. Due to the coexistence of a potentially protective allele and a risk allele that yield conflicting results in the literature, ɛ4/ɛ2 carriers were omitted from the analysis [e.g., 41].

### Memory scores

Repeated cognitive memory composite scores based on the Alzheimer’s Disease Sequencing Project (ADSP) Phenotype Harmonization Consortium were obtained. These composite scores were calculated based on the logical memory subtest (immediate and delayed recall) of the Wechsler Memory Scale Revised, the Rey Auditory Verbal Learning Test, the Alzheimer’s Disease Assessment Scale-Cognitive Subscale (word recall, orientation, and word recognition), the memory-related subtasks of the Mini-Mental State Examination, and the memory-related subtasks of the Montreal Cognitive Assessment. We matched each participant’s neuropsychological assessment timepoint to the closest Aβ-PET scan in time. Further information about the calculation of these composite scores can be retrieved from https://ida.loni.usc.edu/.

### Imaging analysis

FBP-PET and temporally corresponding 3D T1 MRI images were downloaded from ADNI. Additional details of ADNI methods for image acquisition can be obtained from https://adni.loni.usc.edu/methods/.

#### Pre-processing of Aβ-PET images

Global Aβ values were calculated by applying the standard CL approach [37, 42] to the FBP-PET scans via PMOD Neuro Tool (PMOD Technologies LLC). According to the standard protocol, the whole cerebellum was used as a reference area to quantitatively normalize the PET scans. Resulting images were smoothed using a 4-mm full-width at half maximum (FWHM) Gaussian kernel and masked to exclude voxels outside the brain. To convert normalized values into CL units, we used a level-2 calibration for FBP data as described by [43]. The resulting CL values present a single whole brain measure for Aβ load per individual.

For the voxel-wise Aβ analysis, FBP-PET scans were pre-processed with PMOD 4.2 Neuro and Fusion Tool. Each participant’s baseline and last follow-up T1 images were coregistered. Then, the baseline T1 images were normalized using the 6-probability-maps-normalization option. Resulting transformation matrices were applied to the baseline and transformed follow-up FBP-PET images. The normalized PET images were intensity corrected using the cerebellar cortex as reference region and smoothed with a 6-mm FWHM Gaussian kernel. Images were further processed with the Statistical Parametric Mapping software 12 (SPM12; Wellcome Department of Cognitive Neurology, University College, London, UK) running on MATLAB (Mathworks Inc., Natick, MA, USA). Normalized images were visually inspected and coregistered using the SPM template of the common Montreal Neurological Institute (MNI152) reference standard space. Subsequently, annual change rate maps were created for each participant by using the SPM Image Calculator and the formula (Follow-up Scan – Baseline Scan) / Follow-up duration (in years) as proposed by Villain, Chetelat [18]. The resulting annual Aβ change rate maps and the normalized baseline and last follow-up images were smoothed using an isotropic Gaussian filter of 7.155-mm FWHM and were used for further voxel-wise statistical analyses. In the following sections, annual Aβ change rates will be called Aβ accumulation.

#### Pre-processing of T1-weighted MRI images

To investigate anatomical changes, voxel-based morphometry (VBM) was used to examine GM intensity as an atrophy measure. The procedure was adopted for longitudinal data processing using the in SPM12 incorporated option of pairwise longitudinal registration. This tool combines non-linear diffeomorphic and rigid-body registration and corrects for intensity inhomogeneities [44]. All baseline and follow-up scans of the third timepoint of each participant were registered and corrected for bias in an intra-subject manner. As a result, an image of divergences of the longitudinal deformations, Jacobian determinant maps per participant, and a baseline-follow-up average image per participant were created. The baseline-follow-up average images were segmented into tissue classes and used to create a sample-specific template using the SPM Diffeomorphic Anatomical Registration Through Exponentiated Lie Algebra (DARTEL) toolbox [44]. As a next step, this template was normalized using the MNI152 reference standard space. Annual GM atrophy rate maps were created by using SPM Image Calculator and the formula (GM segmentations of the average images * Jacobian maps) / Follow-up duration (in years). The DARTEL generated warps were applied to these atrophy maps. Resulting maps were smoothed with an 8-mm FWHM Gaussian kernel. As a final step, intracranial volume (ICV) was calculated by summating the volumes of GM, white matter, and cerebrospinal fluid based on the SPM tissue segmentations to correct for differences in brain size by integrating it as a covariate into further GM atrophy related statistical analyses. In the following, annual GM atrophy rates will be called GM atrophy.

A sample-specific GM mask was created to later restrict all statistical analyses to GM. In detail, the mask was created using the normalized pre-post average images to calculate a mean image using SPM Image Calculator (based on [45]). This mean image underwent segmentation, generating a GM image that was subsequently thresholded at 0.2. The resulting binary mask underwent manual corrections using the PMOD Fusion Tool to remove any periventricular voxels that were inaccurately classified as GM and was corrected for the basal ganglia and thalamus to prevent partial exclusion of these areas. Finally, the mask was smoothed with a 4-mm FWHM.

### Statistical analysis

R (R version 4.3.3., R Foundation for Statistical Computing, Vienna, Austria, available at https://cran.r-project.org/) was used to compare clinical characteristics of ɛ4 carriers and non-carriers with independent two sample *t*-tests for continuous variables and chi-square tests for categorical variables. Statistical significance for these analyses was set at a *p*-value threshold of less than 0.05.

In order to divide the sample into individuals with and without signs for memory decline over time, we extracted individual memory slopes based on the memory composite scores using the package ‘lme4’ (version 1.1). In detail, we conducted a simple linear mixed regression with time as the only predictor and extracted the random slopes [46–48]. Based on these slopes, all participants with a random slope < 0 were categorized as showing signs of memory decline over time [49]. We assessed the association between memory decline and clinical diagnosis using a chi-square test and a *p*-value threshold of less than 0.05.

Aβ baseline abnormality was determined based on the global CL calculation [37, 42] using a clinical cut-off of 20 CL [50, 51] dividing the sample into participants who had normal Aβ levels (Aβ −) and participants who had already abnormal Aβ levels (Aβ +) at the baseline measurement timepoint.

#### Voxel-wise analysis

To assess voxel-wise interactions separately for each neuroimaging modality and perform multimodal analyses, VoxelStats (v1.1; github.com/sulantha2006/VoxelStats) running on MATLAB was utilized. Simple one-way and two-way voxel-wise *t*-tests were implemented in SPM12. The Automatic Anatomical Labelling 3 (AAL3) atlas was employed to label statistical maps [52]. Visualization was done using the bspmview toolbox (version 20180918) for surface renderings and using the MarsBar toolbox (version 0.45) for region-of-interest (ROI)-based intensity value extractions to create interaction plots and to calculate effect sizes.

##### Aβ progression pattern

To confirm the kml-based classification of Aβ high accumulators and to inspect the general longitudinal Aβ pattern, a one sample *t*-test was applied using the Aβ accumulation maps. Then, to test whether Aβ baseline abnormality should be included as a covariate into further analyses, we assessed the effect of Aβ baseline abnormality on the Aβ accumulation maps with a two-sample *t*-test. The statistical model was adjusted for age, sex, and education.

Subsequently, our primary hypothesis in regard to Aβ accumulation was assessed by two separate ANCOVAs; one to test the main and interaction effects of the APOE genotype and memory decline on Aβ accumulation maps while controlling for clinical diagnosis; and one to test the effects of the APOE genotype and clinical diagnosis on the Aβ accumulation maps while controlling for memory decline. As a sensitivity analysis regarding the inclusion of SMC participants in the MCI group, both ANCOVAs were repeated without SMC participants (Supplementary Table 4). Selective post hoc tests of significant interaction terms were conducted using two-sample *t*-tests. All voxel-wise analyses were additionally adjusted for sex, education, age, and Aβ baseline abnormality, if appropriate. Resulting statistical *t*-maps were thresholded to present significant results with a *p* < 0.001 height, uncorrected for family-wise error (FWE), and a *k* > 100 voxels cluster extent [53]. We additionally remarked those results that survived an even more restrictive *p* < 0.05 FWE corrected threshold. To visualize the results and aid the interpretation, Aβ accumulation values of the significant clusters were extracted using the MarsBar toolbox. These values represent mean intensities of the voxels within the chosen ROI. These Aβ accumulation values were further used to calculate partial eta squared (*η*^2^*p*) as a measure of effect size.

##### Gray matter atrophy pattern

For the investigation of the longitudinal GM atrophy, we applied a similar analysis plan as for the Aβ accumulation pattern. First, a one sample *t*-test was applied to the GM atrophy maps to examine the total atrophy pattern. Then, the effect of Aβ baseline abnormality on GM atrophy maps was tested to determine whether to include Aβ baseline abnormality as a covariate. Our primary hypothesis related to GM atrophy was analyzed by conducting two separate ANCOVAs assessing the main effects and interaction effects of the APOE genotype with memory decline and with clinical diagnosis on the GM atrophy maps, while controlling for the respective other variable. Again, we conducted a sensitivity analysis regarding the inclusion of SMC participants in the MCI group by repeating these two ANCOVAs without SMC individuals (Supplementary Table 4). Selective post hoc tests of interaction terms were conducted using two-sample *t*-tests. The voxel-wise analyses were adjusted for age, sex, education, and total intercranial volume (TIV). Resulting statistical *t*-maps were thresholded to present results with a *p* < 0.001 height, uncorrected for FWE, and a *k* > 100 voxels cluster extent [53] and *p* < 0.05 FWE corrected results were remarked. Subsequently, GM change values of the significant clusters were extracted and visualized to interpret the results. Additionally, they were used to calculate *η*^2^*p*.

##### Multimodal analysis of the Aβ progression and gray matter atrophy pattern

As a final analysis, we tested the association between longitudinal changes in Aβ and GM and the interaction with the APOE genotype and memory decline. We fitted a multimodal linear model via VoxelStats adjusting for Aβ baseline abnormality, age, and TIV. To increase the power of the analysis, we only included the covariates that demonstrated an effect in the previous single modality analyses. Resulting statistical *t*-maps were again thresholded to present results with a *p* < 0.001 height, uncorrected for FWE, and a *k* > 100 voxels cluster extent. We conducted a sensitivity analysis [54] using a less restrictive cluster extent of *k* > 50 voxels [18]. By allowing for the inclusion of these additional voxels, the ROIs incorporated a broader coverage of the relevant area, which might provide a more accurate reflection of the spatial distribution. Within these ROIs, we extracted the mean intensities of included voxels representing Aβ and GM change values. These change values were used to obtain *η*^2^*p*. For visualization purposes, the R package ‘plotly’ (version v2.11.1) was used to create 3D graphs that represent multimodal interactions.

## Results

### Participant characteristics

Demographics, genetic status, and baseline clinical characteristics are displayed in Table 1. Out of 100 participants, 90 entered the voxel-wise Aβ analysis and 85 entered the VBM analysis. The remaining participants had to be excluded from the analysis due to an insufficient image quality for proper pre-processing. Participants were followed over on average 5.71 (*SD* = 1.50) years. A total of 47 participants were classified as APOE ɛ4 carriers and 43 were classified as non-carriers. ɛ4 non-carriers were significantly older and demonstrated lower baseline CL values. The sample was equally divided into 44 Aβ − participants and 46 Aβ + participants. In the ɛ4 carrier group, more participants received an Aβ + status than in the non-carrier group (*X*^*2*^ (1) = 7.53, *p* = 0.01). At baseline, 35 participants were cognitively normal and 55 received an MCI diagnosis. Thirty-nine participants were characterized as having memory decline based on their individual memory slopes, while 51 individuals had no memory decline. Twenty-one participants with memory decline received a baseline diagnosis of MCI. There was no significant association between APOE genotype groups and classification of memory decline, between APOE genotype groups and baseline clinical diagnosis, or between memory decline and baseline clinical diagnosis.

**Table 1:** Clinical characteristics and demographics for APOE ɛ4 carriers and non-carriers

Characteristic	Overall (*n* = 90)	ɛ4 carrier (*n* = 47)	ɛ4 non-carrier (*n* = 43)	Test statistics
Baseline age in years (SD)	72.56 (6.28)	70.52 (6.14)	74.80 (5.67)	*T* = 3.43, *p* < 0.001
Sex (male)	44 (49%)	24 (51%)	23 (53%)	*X*^2^ = 0.19, *p* = 0.67
Diagnosis baseline (CN)	35 (39%)	16 (34%)	19 (44%)	*X*^2^ = 0.97, *p* = 0.32
MMSE baseline (SD)	28.60 (2.08)	28.70 (1.46)	28.49 (2.60)	*T* = − 0.49, *p* = 0.63
Aβ baseline positivity	46 (51%)	30 (64%)	16 (37%)	*X*^2^ = 6.37, *p* = 0.01
Memory decline	39 (43%)	19 (40%)	20 (47%)	*X*^2^ = 0.34, *p* = 0.56
Baseline centiloid (IQR)	20.53 (47.67)	46.16 (58.78)	14.55 (31.30)	*U* = 1484.00, *p* < 0.001
Annual increase in centiloid (SD)	6.45 (3.06)	6.32 (2.97)	6.59 (3.19)	*T* = 0.42, *p* = 0.68
Time interval in years (SD)	5.71 (1.50)	5.70 (1.45)	5.73 (1.57)	*T* = 0.11, *p* = 0.91

*Note*: Independent two sample *t*-tests were used for continuous variables and chi-square tests for categorical variables. *MMSE*, Mini-Mental State Examination; *CN*, cognitively normal; *MCI*, mild cognitive impairment

### Voxel-wise Aβ progression pattern

The voxel-wise one-sample *t*-test of the Aβ accumulation maps revealed a robust and expansive overall Aβ accumulation that reached across the whole brain affecting cortical and subcortical areas (*n* = 90, *t* > 4.42, *p*_FWE_ < 0.05), which supported the CL-based classification of this high accumulator group [19]. The Aβ accumulation was particularly pronounced in the medial orbito-frontal areas, the cingulate cortex, and the precuneus (*n* = 90, *t* > 5.53, *p*_FWE_ < 0.001) (Supplementary Fig. 1).

To test the importance of Aβ baseline abnormality as a covariate, a two-sample *t*-test was used. Baseline Aβ + participants indicated significantly higher accumulation, particularly within the temporal cortex with pronounced changes in the right lobe. Moreover, higher accumulation of Aβ + participants was apparent in parietal and medio-occipital structures, and in the putamen (*n* = 90, *t* > 3.19, *p*_unc_ < 0.001) (Supplementary Fig. 2). Therefore, Aβ baseline abnormality was included as covariate into the subsequent Aβ analyses related to our primary research objective.

**Fig. 1:**
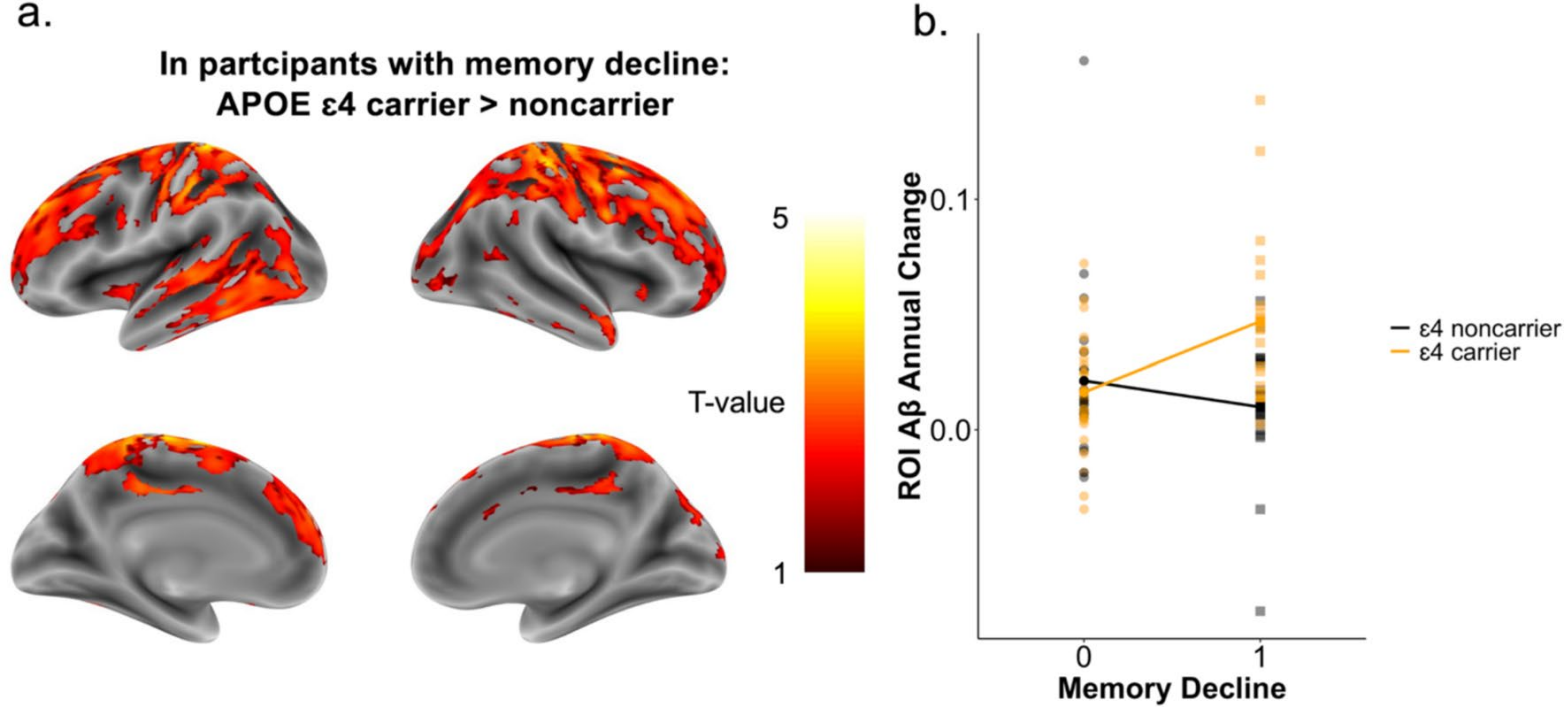
Amyloid beta (Aβ) accumulation dependent on APOE genotype and memory decline. **a** Aβ accumulation pattern in participants with signs of memory decline comparing ɛ4 carriers and non-carriers. Statistical threshold was set to *p*_unc_ < 0.001 and a *k* > 100 voxels cluster extent. Color range displays *T*-values from 1 (red) to 5 (yellow) showing all regions that were significant at a threshold of *p*_unc_ < 0.05 and a *k* > 100 voxels cluster extent. **b** Visualization of the interaction of APOE genotype and signs of memory decline (memory decline = 1) on region-of-interest (ROI)-based mean intensities of annual Aβ change rates in pre- and post-central areas, and the superior frontal gyrus

A two-way ANCOVA was applied to compare the effect of APOE genotype groups, memory decline groups, and their interaction on Aβ accumulation maps independent of baseline clinical diagnosis. We observed a significant voxel-wise interaction of genotype and memory decline in the area around the right paracentral lobule (*n* = 90, *t* = 3.81, *p*_unc_ < 0.001, *η*^2^*p* = 0.11). No main effects of APOE genotype or memory decline were observed. Post hoc tests confirmed that ɛ4 carriers with signs of memory decline (*n* = 19) had significantly more Aβ accumulation in the pre- and post-central areas and in the left superior frontal gyrus (*t* > 3.36, *p*_unc_ < 0.001) in comparison to non-carriers with signs of memory decline and revealed a trend for additionally more accumulation of ɛ4 carriers in other frontal areas, in temporal areas, and in the putamen (*t* > 1.69, *p*_unc_ < 0.05) (Fig. 1, Supplementary Table 1). In participants without signs of memory decline, no such difference was revealed. To confirm that this result was not solely driven by Aβ + individuals, we subsequently conducted the two-way ANCOVA in only Aβ − individuals (*n* = 44). The results indicated a similar trend at *p*_unc_ < 0.05 and a *k* > 100 voxels cluster extent (Supplementary Fig. 6A), despite the smaller sample size. Thus, these results might suggest that early processes of Aβ accumulation may differ in APOE ε4 carriers with memory decline—even when global Aβ levels are not yet elevated.

A second ANCOVA compared the main and interaction effects of the baseline clinical groups and the APOE genotype on Aβ accumulation independent of memory decline. No significant interaction or main effect was found. That is, while APOE genotype appeared to have an association with Aβ accumulation in the context of memory decline, this relationship was not evident when considering clinical diagnosis categories of CN and MCI independently of memory decline.

### Voxel-wise gray matter atrophy pattern

Overall, the high accumulator group under investigation demonstrated significant GM atrophy across the whole cortex, especially pronounced in temporal areas, in the left precuneus, and the left thalamus (*n* = 85, *t* > 4.66, *p*_FWE_ < 0.05) (Supplementary Fig. 3).

To determine if Aβ baseline abnormality should be included as a covariate in further analyses, we tested its effect on GM atrophy. No significant differences between baseline Aβ − and Aβ + groups on GM atrophy were observed, and therefore the variable was not included as covariate into further GM atrophy analyses related to our primary research question.

**Fig. 2:**
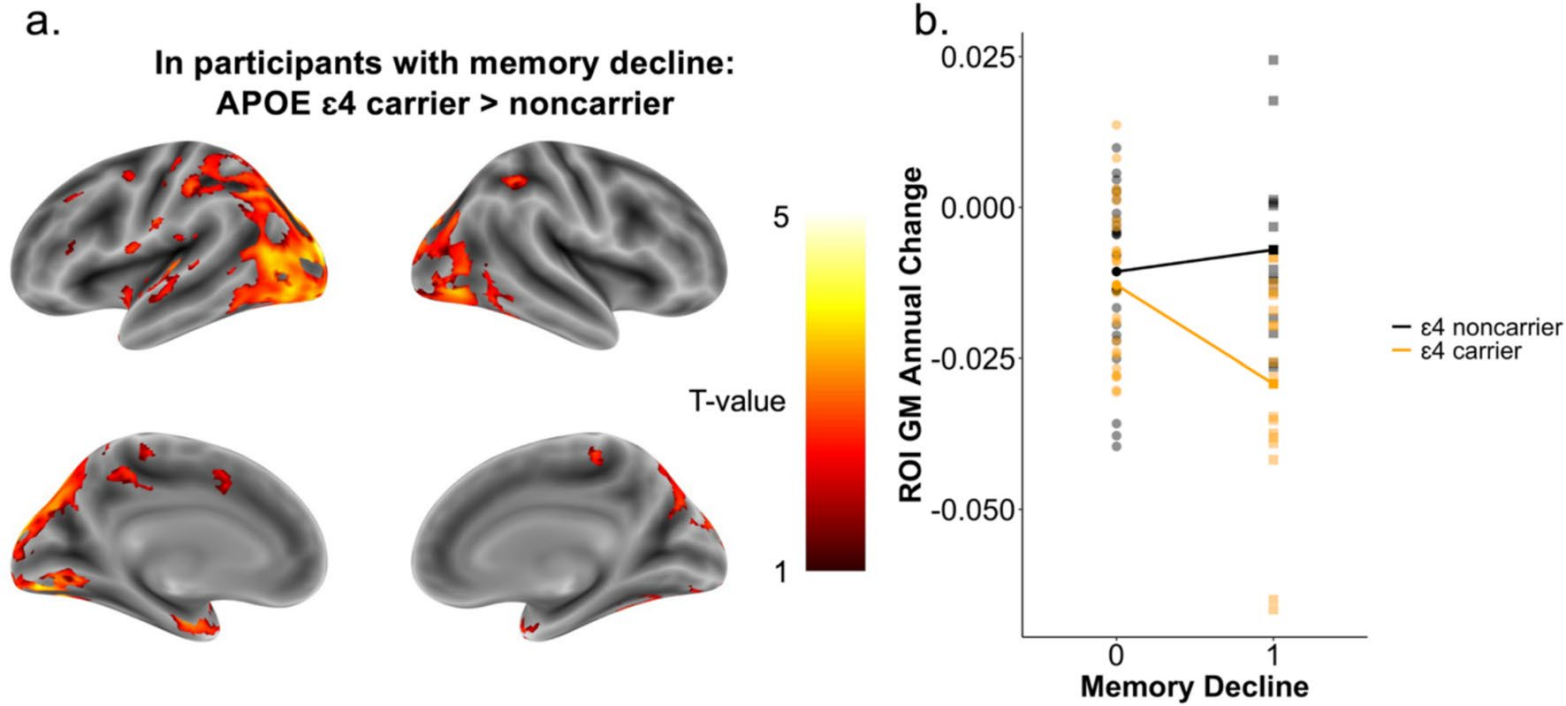
Gray matter (GM) atrophy dependent on APOE genotype and memory decline. **a** Gray matter (GM) atrophy pattern over time in participants with memory decline comparing ɛ4 carriers to non-carriers. Statistical threshold was set to *p*_unc_ < 0.001 and a *k* > 100 voxels cluster extent. Color range displays *T*-values from 1 (red) to 5 (yellow) showing all regions that were significant at a threshold of *p*_unc_ < 0.05 and a *k* > 100 voxels cluster extent. **b** Visualization of the interaction of APOE genotype and signs of memory decline (memory decline = 1) on region-of-interest (ROI)-based mean intensities of annual GM change rates in occipital areas

We performed a two-way ANCOVA to compare how the APOE genotype and memory decline groups, and the combination of both, affected the GM atrophy. The results demonstrated a significant interaction between the APOE genotype and memory decline in left medio-temporal areas surrounding the parahippocampal gyrus, the left temporal pole, and the left occipital cortex (*t* > 3.20, *p*_unc_ < 0.001, *η*^2^*p* = 0.15). Genotype and memory decline groups revealed no main effects on GM atrophy. Post hoc tests showed that among participants with memory decline, carriers (*n* = 18) exhibited significantly more atrophy than non-carriers (*n* = 14) in the occipital cortex (*t* > 3.44, *p*_unc_ < 0.001) and revealed a trend for a more widespread atrophy pattern in several other areas such as the left hippocampus, the left lingual gyrus, the pre- and post-central areas, and some frontal areas (*t* > 1.71, *p*_unc_ < 0.05, Fig. 2, Supplementary Table 2). In participants with no memory decline, we observed no significant differences between genotypes. We confirmed that the interaction was independent of Aβ baseline abnormality by repeating the two-way ANCOVA in only Aβ − individuals (*n* = 42) demonstrating similar regional trends (Supplementary Fig. 6B). Similar to the results of Aβ accumulation, these findings might suggest that early processes related to gray matter atrophy might be different in APOE ε4 carriers with memory decline.

The second ANCOVA revealed a significant main effect of the baseline clinical group on GM atrophy at *p*_unc_ < 0.001. MCI participants displayed higher GM atrophy in the areas around the right superior temporal gyrus (*t* = 3.81, *p*_unc_ < 0.001) and the right olfactory cortex (*t* = 3.65, *p*_unc_ < 0.001) in comparison to CN participants. The interaction of baseline clinical group and APOE genotype was not significant.

### Multimodal voxel-wise results

**Fig. 3:**
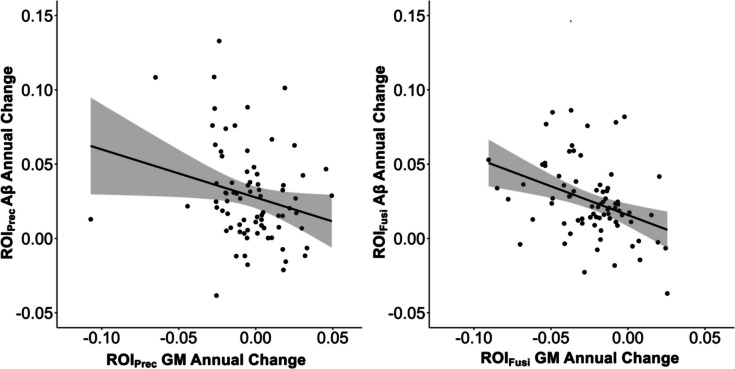
Multimodal association of amyloid beta (Aβ) accumulation and gray matter (GM) atrophy. Scatterplot of the association between Aβ annual change and gray matter (GM) change for the region-of-interests (ROIs) based on the significant areas surrounding the precentral gyrus and the fusiform/occipital cortex extracted from the voxel-wise analysis of the three-way interaction of GM atrophy, APOE genotype, and memory decline on Aβ accumulation. Both scatterplots visualize that more GM atrophy is related to higher Aβ accumulation

The multimodal linear regression revealed a significant three-way interaction between APOE genotype, memory decline, and GM atrophy on Aβ accumulation in the areas around the fusiform gyrus of both hemispheres (*t* > 3.22, *p*_unc_ < 0.001, *k* > 100). We conducted a sensitivity analysis [54] using a less restrictive cluster extent of *k* > 50 voxels [18]. This threshold confirmed the significance of the multimodal association in the areas around the fusiform gyrus. Additionally, the less restrictive threshold revealed the right inferior occipital cortex and the left precentral gyrus to indicate the three-way relationship. However, we observed that the result of the fusiform and occipital cortex (“positive contrast”) differed in direction compared to that of the precentral gyrus (“negative contrast”) (Supplementary Table 3). This dissimilarity indicates a divergence in the meaning of the interaction and the relationship of the groups with each other. To further explore this relationship, two separate ROIs were created; one for the area surrounding the fusiform gyrus and the occipital cortex (ROI_Fusi_) and one for the area surrounding the precentral gyrus (ROI_Prec_). These ROIs were based on the significant voxel-wise clusters and the extracted values represented the mean intensities of Aβ accumulation and GM atrophy maps. Generally, GM change and Aβ change were significantly correlated in both ROIs (*r*_Fusi_ = − 0.37, *p*_Fusi_ < 0.001; *r*_Prec_ = − 0.24, *p*_Prec_ = 0.039) (Fig. 3). Notably, the ROI_Fusi_ displayed overall more atrophy than the ROI_Prec_ (*V* = 448, *p* < 0.001). Repeating the multimodal linear regression with the extracted change values separately for each ROI confirmed the three-way interaction (*β*_Fusi_ = 1.90, *p*_Fusi_ < 0.001, *η*^2^*p*_Fusi_ = 0.19; (*β*_Prec_ = − 2.04, *p*_Prec_ = 0.002, *η*^2^*p*_Prec_ = 0.14) (Supplementary Figs. 4 and 5 display 3D plots that visualize the complex relationships). In detail, based on the ROI_Fusi_ values, ɛ4 non-carriers with atrophy and memory decline displayed the highest Aβ accumulation (Supplementary Fig. 4). ɛ4 carriers had a higher accumulation than non-carriers in individuals with atrophy, but no signs of memory decline (Supplementary Fig. 4). Conversely, based on ROI_Prec_ values, ɛ4 carriers with atrophy and signs of memory decline showed the highest Aβ accumulation (Supplementary Fig. 5). Nonetheless, these interpretations should be considered with caution due to the limited cluster extent and the use of extracted mean intensities. To confirm that the three-way interaction was not solely due to Aβ + individuals, we additionally conducted the multimodal linear regression in only Aβ − individuals (*n* = 36). The results indicated a similar trend at *p*_unc_ < 0.05 and a *k* > 100 voxels cluster extent (Supplementary Fig. 6C).

## Discussion

The current investigation focused on a relevant subgroup of older adults who exhibited significant increases in global Aβ burden over time but did not receive a dementia diagnosis yet. Evidence suggests that the extraction of Aβ accumulators is highly relevant for predicting potential AD disease courses and downstream pathologies [21–24] and might represent an early disease stage [20, 23, 24]. Within this subgroup, we investigated the regional progression pattern of Aβ and GM atrophy, along with its potential dependency on the APOE genotype. A crucial aspect of the current analysis was considering the additional relation with dynamic memory decline in comparison to baseline clinical diagnosis, while controlling for Aβ baseline abnormality.

The primary finding of the present study was the detection of an interaction between the APOE genotype and memory decline on the voxel-wise change rates of both Aβ and GM atrophy. In detail, ɛ4 carriers with declining memory scores over time demonstrated significantly more Aβ accumulation in comparison to non-carriers with memory decline in predominantly pre- and post-central areas (Fig. 1). Concurrently, ɛ4 carriers demonstrated more GM atrophy in the occipital cortex, in predominantly left medio-temporal areas, and in the left temporal pole (Fig. 2). This difference between genotypes was not observable in the memory stable group. In accordance with the present results, previous studies have reported a possible interplay between APOE genotype, memory decline, and abnormal baseline Aβ levels [30, 31, 55]. For example, Lim, Villemagne [31] investigated healthy older adults and observed a faster memory decline in baseline Aβ abnormal ɛ4 carriers. Another study examining healthy middle-aged participants observed that cognitively unimpaired ɛ4 carriers with Aβ abnormality in specific brain regions, such as temporal and parietal areas, and the striatum displayed increased cognitive decline [30]. However, these studies did not fully explore the relation with longitudinal Aβ accumulation, which was the focus of our investigation, and were based on global or selected regional Aβ measures. Accordingly, by examining the annual change rates of Aβ in a voxel-wise manner, the present study identified different areas, especially in the sensorimotor cortex, that displayed the interactive effect. Regarding GM atrophy, the observed interaction between the APOE genotype and memory decline might provide a potential explanation for previously conflicting results concerning the link between GM atrophy and APOE genotype. Several prior studies investigating the association of increased longitudinal GM atrophy and ɛ4 carriers did not account for dynamic memory decline [16, 27–29]. Moreover, our finding accords with one previous study that detected a similar interplay [32]. Specifically, Haller, Montandon [32] suggested that APOE genotype was solely related to posterior cingulate cortex GM atrophy in healthy individuals who exhibited cognitive decline. However, the researchers found no such interaction in other regions. Our analysis identified a broader range of regions, particularly including the left medio-temporal areas surrounding the parahippocampal gyrus, the left temporal pole, and the left occipital cortex, that demonstrated more GM atrophy in ɛ4 carriers with signs of memory decline. Although Haller, Montandon [32] considered the longitudinal aspect of cognitive decline, they used baseline MRI scans to compare GM atrophy between groups. As for Aβ, our voxel-wise analysis considered the longitudinal change of GM in terms of annual change rates, which might lead to the difference in the observed regional involvement. Importantly, in our study, participants showing memory decline without the ɛ4 allele did not have significantly increased Aβ accumulation or GM atrophy rates in any region. Likewise, no significant genotype difference was observed within the memory stable group. Together, the findings might suggest a distinct spatiotemporal biomarker pattern of ɛ4 carriers with memory decline. In a clinical context, these results might suggest that early cognitive complaints in ε4 carriers need to be closely monitored in terms of subtle memory decline, as this might be already an indication of underlying pathological changes associated with AD. Moreover, these results underscore the potential benefit of initiating earlier intervention within these individuals to mitigate or slow further disease progression.

The goal of the final analysis was to examine the relation between the two AD pathologies in more detail. The multimodal analysis detected evidence for a three-way interaction of APOE genotype, memory decline, and longitudinal GM atrophy, which are collectively related to Aβ accumulation in the areas surrounding the fusiform gyrus. The application of a less restrictive cluster extent threshold (*k* > 50 voxels) revealed the possible relevance of additional regions, specifically the right inferior occipital cortex and the left precentral gyrus, which also exhibited this three-way relationship. However, there was a directional divergence in the relationship between the fusiform and occipital regions (positive contrast) and the precentral area (negative contrast). The areas around the fusiform and occipital cortex indicated that ɛ4 non-carriers with both atrophy and memory decline displayed the highest Aβ accumulation. ɛ4 carriers seemed to show more accumulation in individuals who exhibited atrophy but no signs of memory decline. In contrast, the precentral gyrus displayed the highest Aβ accumulation in participants with ɛ4 carrier status, atrophy, and memory decline. This divergence might result from the distinct temporal involvement of the different brain regions and the two AD pathologies during the disease course. Atrophy in the fusiform gyrus and in the occipital cortex has been observed early during the disease course [56, 57]. The current results suggest that these regions might show pathological alterations and resulting multimodal associations either in ɛ4 carriers who have not yet experienced memory decline, or in ɛ4 non-carriers who already present with memory decline. Considering the additional knowledge that carriage of an ɛ4 allele shifts the onset of Aβ deposition to an earlier time point [e.g., 21, 58], our results might further emphasize an early-stage disease involvement of these regions. Conversely, sensorimotor regions, such as the precentral gyrus, are typically affected during later AD stages in regard to Aβ accumulation [7, 53, 59]. The current results are in line with this later stage involvement as they indicate that the precentral areas present the multimodal association in ɛ4 carriers who already experienced memory decline. Further in accordance with our multimodal findings, a previous study investigating the voxel-wise relation between cross-sectional data of GM and Aβ-PET data in AD patients also reported an association between the neuroimaging modalities in the fusiform gyrus [60]. However, the researchers neither considered the APOE genotype nor cognitive decline. Two other studies attempted to investigate the inter-relations of Aβ levels, structural changes, the APOE genotype, and cognitive decline. Li, Loewenstein [61] used a region-of-interest approach and evaluated the independent and combined effects of Aβ abnormality and APOE genotype on cortical thinning and cognition in CN, MCI, and AD participants of the ADNI cohort. They reported independent effects of Aβ abnormality and APOE genotype on cortical thinning and cognitive impairment in late MCI and AD patients. Conversely, in CN and early MCI participants, they observed cortical thickening. Combined effects of genotype and Aβ abnormality were only observed for cognition. However, they did not analyze structural changes and cognitive impairments within one statistical model, and the researchers did not investigate changes over time. The other study targeting the inter-relations was a longitudinal study investigating the association of hippocampal volume loss, APOE genotype, cognitive decline, and Aβ status in CN participants [62]. The investigation demonstrated independent effects of cognitive decline and APOE genotype on hippocampal volume loss but found no association of Aβ status and hippocampal volume and no interaction. In contrast to our analysis, the research did not model the interaction of cognitive decline and APOE and only included Aβ abnormality. In sum, these findings underscore the complexity of the relation between the AD pathologies and the APOE genotype. The multimodal associations further confirm that the spatiotemporal biomarker patterns vary between ɛ4 carriers and non-carriers. In practice, these findings further emphasize the importance of closely monitoring ɛ4 carriers for early signs of memory decline as this might already indicate pathological disease progression.

An interesting observation was that memory decline, as defined by the reduction of neuropsychological scores over time, appeared to be distinct from the clinical categorization of MCI. Our analysis found no association between the group of participants that had signs of longitudinal memory decline and the group of participants that received an MCI diagnosis at the baseline measurement time point. Moreover, we observed no interaction of baseline clinical group and the APOE genotype in terms of Aβ accumulation or GM atrophy. Together with previous findings on the relevance of memory decline [30–32, 55, 62], our observations might implicate that categorizing patients based on their ongoing decline in cognitive scores over time might be more informative than the static classification as CN or MCI in regard to ongoing AD-related pathological progression patterns. Thus, this observation further highlights the importance of repetitive neuropsychological assessments for a comprehensive understanding of the disease progression and proper individual predictions. Moreover, these findings encourage future studies to further identify factors that define individuals with memory decline. Such knowledge might refine clinical trial design by enabling researchers to better target high-risk populations, thereby improving the likelihood of demonstrating a treatment effect and increasing the potential for earlier, more effective interventions.

Last, our voxel-wise investigation reinforces the value of focusing on the high Aβ accumulators representing an early AD disease stage as they displayed extensive and regionally widespread Aβ accumulation and GM atrophy patterns over time closely aligning with patterns observed in later AD stages [18, 53, 63]. That is, Aβ accumulation was especially dominant in areas of the medial orbito-frontal cortex, the cingulate cortex, and the precuneus, while longitudinal GM atrophy was pronounced in temporal, occipital, orbito-frontal, and parietal cortical areas. Moreover, the current investigation revealed accelerated atrophy rates in the thalamus. A recent cross-sectional examination based on ADNI data proposed that individuals converting to AD exhibited GM loss in similar areas such as mid-temporal areas, the posterior thalamus, and the angular gyrus at baseline [13] suggesting a possible conversion risk for our subsample. The multimodal analysis which was able to demonstrate an association between Aβ accumulation and GM atrophy while still indicating spatiotemporal divergences might recommend these high Aβ accumulators as important group when studying the early associations between the AD biomarkers. Furthermore, our examination strengthens the idea of “high accumulators” as important candidates for future clinical trials for disease-modifying therapies [20, 21]. Specifically, since the recently approved disease-modifying AD treatments by the US Food and Drug Administration (FDA) and European Medicines Agency (EMA) target brain Aβ removal to slow disease progression, but cannot halt or reverse cognitive impairment [51, 64, 65], this stage might offer a critical and early intervention window. Therefore, a better understanding of this group and the identification of synergistic risk factors is highly needed to further optimize clinical trial designs and to refine early intervention strategies.

The current study had some limitations. The investigation used data obtained from the multicenter ADNI cohort that may introduce several methodological variabilities as participants were assessed on different scanner types varying between study centers and across time points. The variance was minimized by careful pre-processing and repeated visual inspections. Additionally, the current focus on the high accumulator subsample led to a relatively small sample size and could introduce selection biases, since it specifically targets individuals who already show a steep trajectory of amyloid accumulation. Consequently, these findings may not fully generalize to individuals with stable or minimal amyloid increases, and the observed APOE or cognitive effects might be amplified relative to a more heterogeneous cohort. Nonetheless, focusing on this group was beneficial in comparison to other investigations, as it offers a unique group of individuals within an early and critical window of ongoing pathology accumulation. Thus, the subsample under investigation provided valuable information about the dynamic course of pathology. A comparison to low or medium accumulators and non-accumulators might be of heightened interest for future research to receive additional insights into differences in baseline characteristics or risk factors and early disease processes. Another limitation concerns the definition of memory decline based on random slopes, which is not a clinical definition. However, this definition represents an accepted statistical approach that has previously been used with evidence suggesting its clinical relevance [66]. By including all individuals who displayed decreases in memory scores over time, we aimed to capture even subtle differences. A major strength of the current investigation was the analysis of longitudinal data that encompass a rather long time interval of on average 6 years in comparison to previous examinations ([e.g., 53, 67]).

## Conclusion

The current investigation demonstrated the influence of the APOE ɛ4 allele on the progression of Aβ accumulation and GM atrophy, presenting evidence for a distinct spatiotemporal pattern in individuals with memory decline. These results not only advance our knowledge of biomarker dynamics but might facilitate the refinement of early intervention strategies. Moreover, the results might emphasize the need for early and repeated assessments of memory performance among ε4 carriers to improve the detection of possible pathological brain alterations and disease progression.

## Supplementary Information

Below is the link to the electronic supplementary material.

Supplementary file1 (DOCX 39753 KB)

## Data Availability

The ADNI data are available to the scientific community without embargo at http://adni.loni.usc.edu/data-samples/access-data/ after approval by the Data Sharing and Publications Committee and adherence to the ADNI Data Use Agreement and publication policies.
