# Supplementary material for: Voxel-wise insights into early Alzheimer’s disease pathology progression: the association with APOE and memory decline
Source: GeroScience. 2025 Apr 1;47(4):5825–42. doi: 10.1007/s11357-025-01610-z (PMC12397024; doi:10.1007/s11357-025-01610-z)
Supplement: Supplementary file 1 — Supplementary file1 (DOCX 39753 KB) [file 11357_2025_1610_MOESM1_ESM.docx]

**Voxel-wise insights into early Alzheimer’s disease pathology progression: the association with APOE and memory decline**

GeroScience

Maha Wybitul, Nicolas Langer, Christoph Hock, Anton Gietl, Valerie Treyer, for the Alzheimer’s Disease Neuroimaging Initiative

**Corresponding Author:** Valerie Treyer, valerie.treyer@usz.ch, Institute for Regenerative Medicine, Faculty of Medicine, University of Zurich, 8952 Schlieren; Department of Nuclear Medicine, University of Zurich, 8091 Zurich, Switzerland

**Supplementary material**

**Supplementary Tables**

**Supplementary Table 1** In participants with memory decline, two-sample t-test on Aβ change maps: carriers > noncarriers

| Contrast Name | |  |  | MNI Coordinates | | |
| --- | --- | --- | --- | --- | --- | --- |
|  | Region Label | Extent | t-value | x | y | z |
|  |  |  |  |  |  |  |
| Positive | Frontal_Sup_2_L | 74550 | 4.154 | -16 | -5 | 71 |
|  | Paracentral_Lobule_R | 74550 | 4.143 | 1 | -28 | 73 |
|  | Postcentral_R | 74550 | 3.747 | 41 | -32 | 62 |
|  | Temporal_Pole_Mid_R | 918 | 2.660 | 52 | 7 | -25 |
|  | Temporal_Inf_R | 918 | 2.140 | 52 | -21 | -22 |
|  | Putamen_R | 839 | 2.606 | 26 | 14 | -5 |
|  | Putamen_R | 839 | 1.924 | 24 | -1 | 8 |
|  | Temporal_Mid_R | 570 | 1.895 | 52 | -46 | -7 |
|  | Temporal_Mid_R | 570 | 1.837 | 49 | -55 | 11 |
|  | Temporal_Inf_R | 570 | 1.734 | 60 | -32 | -25 |

*Note.* t > 1.6924; p < 0.05; df = 33; minimum extent = 100. Table shows all local maxima separated by more than 20 mm. Regions were automatically labeled using the Automatic Anatomical Labelling atlas 3 (AAL3). x, y, and z = Montreal Neurological Institute (MNI) coordinates in the left-right, anterior-posterior, and inferior-superior dimensions, respectively.

**Supplementary Table 2** In participants with memory decline, two-sample t-test on GM change maps: carriers > noncarriers

| Contrast Name | |  |  | MNI Coordinates | | |
| --- | --- | --- | --- | --- | --- | --- |
|  | Region Label | Extent | t-value | x | y | z |
|  |  |  |  |  |  |  |
| Positive | Occipital_Sup_L | 32915 | 5.102 | -18 | -93 | 20 |
|  | Lingual_L | 32915 | 4.162 | -11 | -83 | -11 |
|  | Occipital_Mid_L | 32915 | 3.906 | -41 | -71 | 8 |
|  | Hippocampus_L | 4999 | 3.654 | -26 | -14 | -24 |
|  | Temporal_Sup_L | 4999 | 3.126 | -45 | -24 | 6 |
|  | Temporal_Pole_Sup_L | 4999 | 3.042 | -26 | 9 | -32 |
|  | Postcentral_L | 250 | 3.223 | -63 | -6 | 24 |
|  | ParaHippocampal_R | 1662 | 2.775 | 21 | 5 | -30 |
|  | ParaHippocampal_R | 1662 | 2.375 | 30 | -15 | -24 |
|  | Hippocampus_R | 1662 | 2.212 | 29 | -30 | -11 |
|  | Cerebellum_7b_L | 220 | 2.753 | -45 | -60 | -53 |
|  | Frontal_Mid_2_L | 241 | 2.731 | -32 | 32 | 45 |
|  | Cerebellum_Crus2_R | 1038 | 2.719 | 9 | -83 | -44 |
|  | Cerebellum_Crus2_R | 1038 | 2.177 | 44 | -77 | -38 |
|  | Paracentral_Lobule_R | 293 | 2.643 | 8 | -35 | 51 |
|  | Cerebellum_Crus2_L | 101 | 2.604 | -45 | -74 | -42 |
|  | Cingulate_Mid_L | 514 | 2.506 | -9 | -38 | 45 |
|  | Frontal_Inf_Tri_L | 113 | 2.328 | -47 | 30 | 14 |
|  | SupraMarginal_L | 136 | 2.271 | -44 | -32 | 24 |
|  | Cerebellum_9_L | 313 | 2.163 | -3 | -54 | -35 |
|  | Cingulate_Mid_L | 127 | 2.068 | -11 | 5 | 44 |
|  | Precentral_L | 196 | 2.055 | -39 | -2 | 48 |

*Note.* t > 1.7056; p < 0.05; df = 26; minimum extent = 100. Table shows all local maxima separated by more than 20 mm. Regions were automatically labeled using the Automatic Anatomical Labelling atlas 3 (AAL3). x, y, and z = Montreal Neurological Institute (MNI) coordinates in the left-right, anterior-posterior, and inferior-superior dimensions, respectively.

**Supplementary Table 3** Multimodal three-way interaction

| Contrast Name | |  |  | MNI Coordinates | | |
| --- | --- | --- | --- | --- | --- | --- |
|  | Region Label | Extent | t-value | x | y | z |
|  |  |  |  |  |  |  |
| Positive | Fusiform_L | 245 | 4.086 | -32 | -18 | -30 |
|  | Occipital_Inf_R | 67 | 3.963 | 38 | -87 | 0 |
|  | Fusiform_R | 118 | 3.839 | 21 | 6 | -44 |
| Negative | Precentral_L | 68 | -3.890 | -39 | -3 | 59 |

*Note.* t > 3.2145; p < 0.001; df = 68; minimum extent = 50. Table shows all local maxima separated by more than 20 mm. Regions were automatically labeled using the Automatic Anatomical Labelling atlas 3 (AAL3). x, y, and z = Montreal Neurological Institute (MNI) coordinates in the left-right, anterior-posterior, and inferior-superior dimensions, respectively.

**Supplementary Table 4** Sensitivity analysis excluding participants characterized as having subjective memory complaints (SCM) (n = 13)

| **Model: Amyloid change maps = APOE genotype * Memory decline + Baseline Diagnosis + Amyloid Abnormality + Age + Sex + Education (df = 68)** | | | | | | |
| --- | --- | --- | --- | --- | --- | --- |
| Effect | |  |  | MNI Coordinates | | |
|  | Region Label | Extent | t-value | x | y | z |
|  |  |  |  |  |  |  |
| APOE genotype * Memory decline | Paracentral_Lobule_R | 80 | 3.423 | 5 | -32 | 75 |
| **Model: GM atrophy = APOE Genotype * Memory decline + Baseline Diagnosis + Age + Sex + Education + TIV (df = 62)** | | | | | | |
| Effect | |  |  | MNI Coordinates | | |
|  | Region Label | Extent | t-value | x | y | z |
|  |  |  |  |  |  |  |
| APOE genotype * Memory decline | Occipital_Inf_L | 538 | -3.707 | -41 | -77 | -8 |
|  | Amygdala_L | 93 | -3.934 | -24 | -5 | -14 |
|  | Temporal_Pole_Sup_L | 67 | -3.894 | -24 | 11 | -33 |
|  | ParaHippocampal_L | 87 | -3.894 | -27 | -14 | -26 |
|  | Occipital_Inf_L | 538 | -3.707 | -41 | -77 | -8 |
| Memory decline | Pallidum_R | 360 | -4.416 | 26 | -2 | -5 |
|  | SupraMarginal_R | 108 | -3.762 | 56 | -36 | 35 |
| **Model: GM atrophy = APOE Genotype * Baseline Diagnosis + Memory decline + Age + Sex + Education + TIV (df = 62)** | | | | | | |
| Effect | |  |  | MNI Coordinates | | |
|  | Region Label | Extent | t-value | x | y | z |
|  |  |  |  |  |  |  |
| Baseline Diagnosis | Temporal_Sup_R | 309 | 4.131 | 62 | -35 | 6 |
|  | Temporal_Pole_Sup_R | 85 | 3.935 | 50 | 18 | -18 |
|  | Temporal_Sup_R | 84 | 3.864 | 60 | -8 | -8 |
|  | Frontal_Inf_Orb_2_R | 56 | 3.564 | 36 | 27 | -11 |
| APOE genotype | Frontal_Inf_Orb_2_R | 166 | 4.210 | 36 | 27 | -6 |
|  | Temporal_Sup_R | 56 | 3.816 | 56 | -36 | 9 |

*Note.* The four different main models have been tested excluding the participants with SMC: (1) Model 1: Amyloid change maps = APOE genotype * Memory decline + Baseline Diagnosis + Amyloid Abnormality + Age + Sex + Education; (2) Model 2: Amyloid change maps = APOE genotype * Baseline Diagnosis + Memory decline + Amyloid Abnormality + Age + Sex + Education; (3) GM atrophy = APOE Genotype * Memory decline + Baseline Diagnosis + Age + Sex + Education + TIV; (4) GM atrophy = APOE Genotype * Baseline Diagnosis + Memory decline + Age + Sex + Education + TIV. The table displays only the significant regions at p < 0.001; k > 50. The table shows all local maxima separated by more than 20 mm. Regions were automatically labeled using the Automatic Anatomical Labelling atlas 3 (AAL3). x, y, and z = Montreal Neurological Institute (MNI) coordinates in the left-right, anterior-posterior, and inferior-superior dimensions, respectively. Despite the reduced sample size, the key findings remained largely consistent, involving similar brain regions and effect patterns.

**Supplementary Figures**


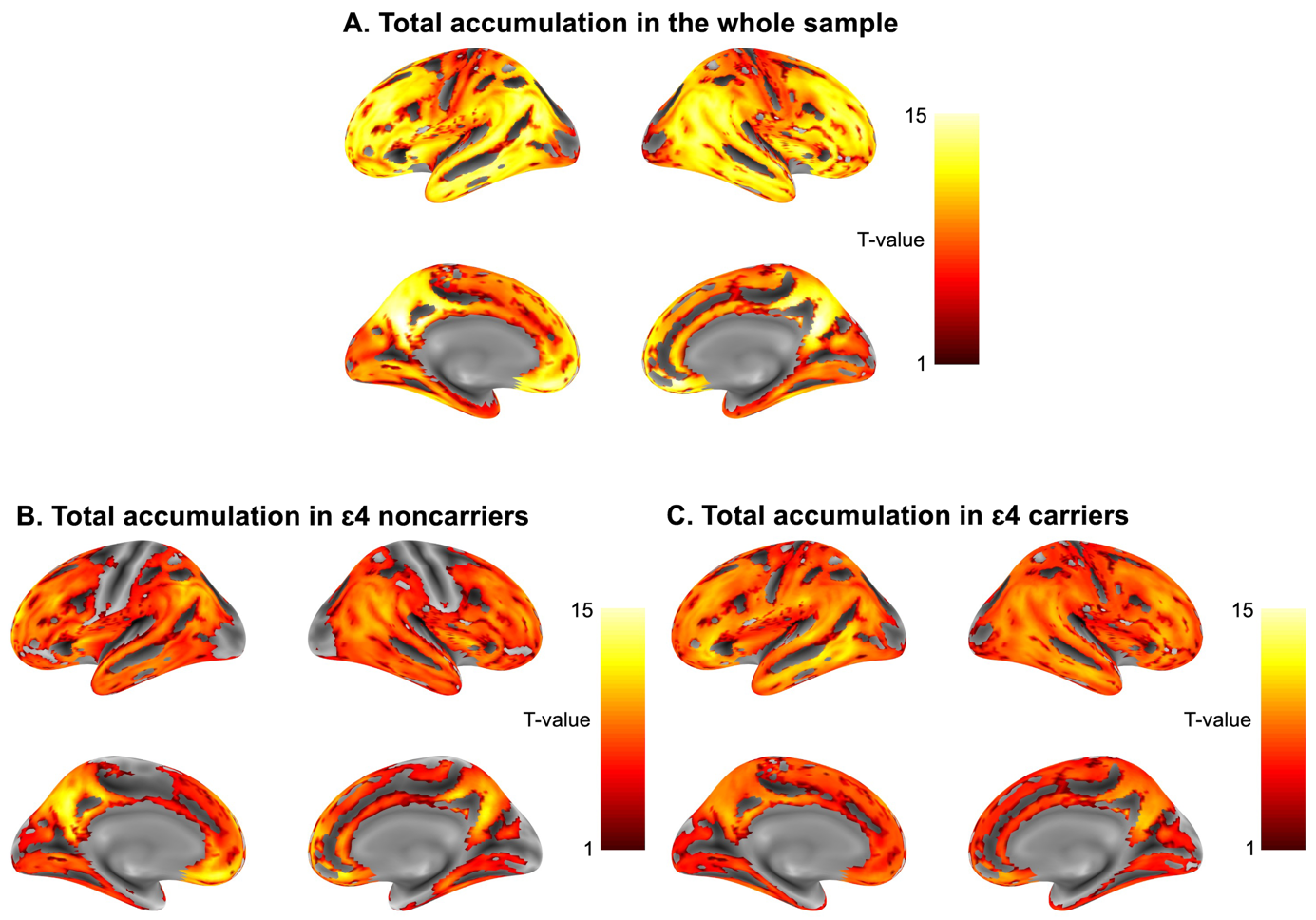


**Supplementary Fig. 1** Total Aβ accumulation **(**A) Aβ accumulation in the whole sample particularly pronounced in the medial orbito-frontal areas, the cingulate cortex, and the precuneus. (B) Aβ accumulation in ɛ4 noncarriers. (C) Aβ accumulation in ɛ4 carriers. Statistical threshold was set to *p_FWE_* < 0.05 and a k > 100 voxels cluster extent. Color range displays T-values from 1 (red) to 15 (yellow) showing all significant regions


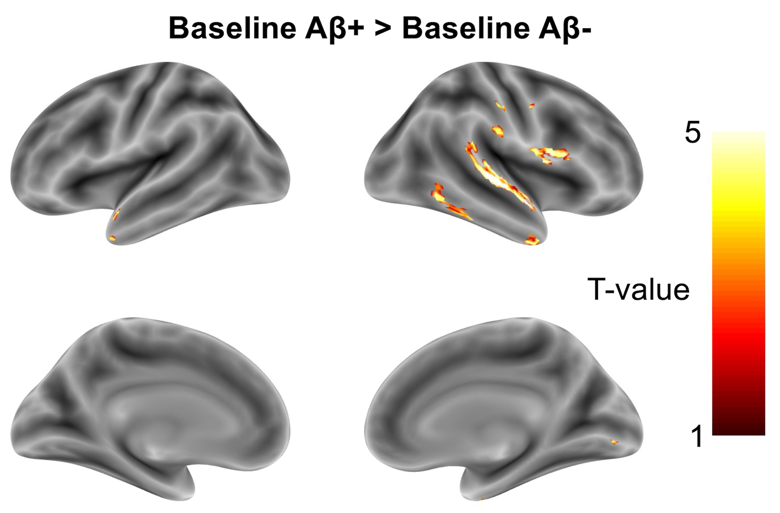


**Supplementary Fig. 2** Dependence of amyloid beta (Aβ) accumulation on baseline Aβ abnormality. Aβ accumulation comparing baseline amyloid beta normal (Aβ-) and baseline amyloid beta abnormal (Aβ+) individuals. Statistical threshold was set to *p_unc_* < 0.001 and a k > 100 voxels cluster extent. Color range displays T-values from 1 (red) to 5 (yellow) showing all significant regions


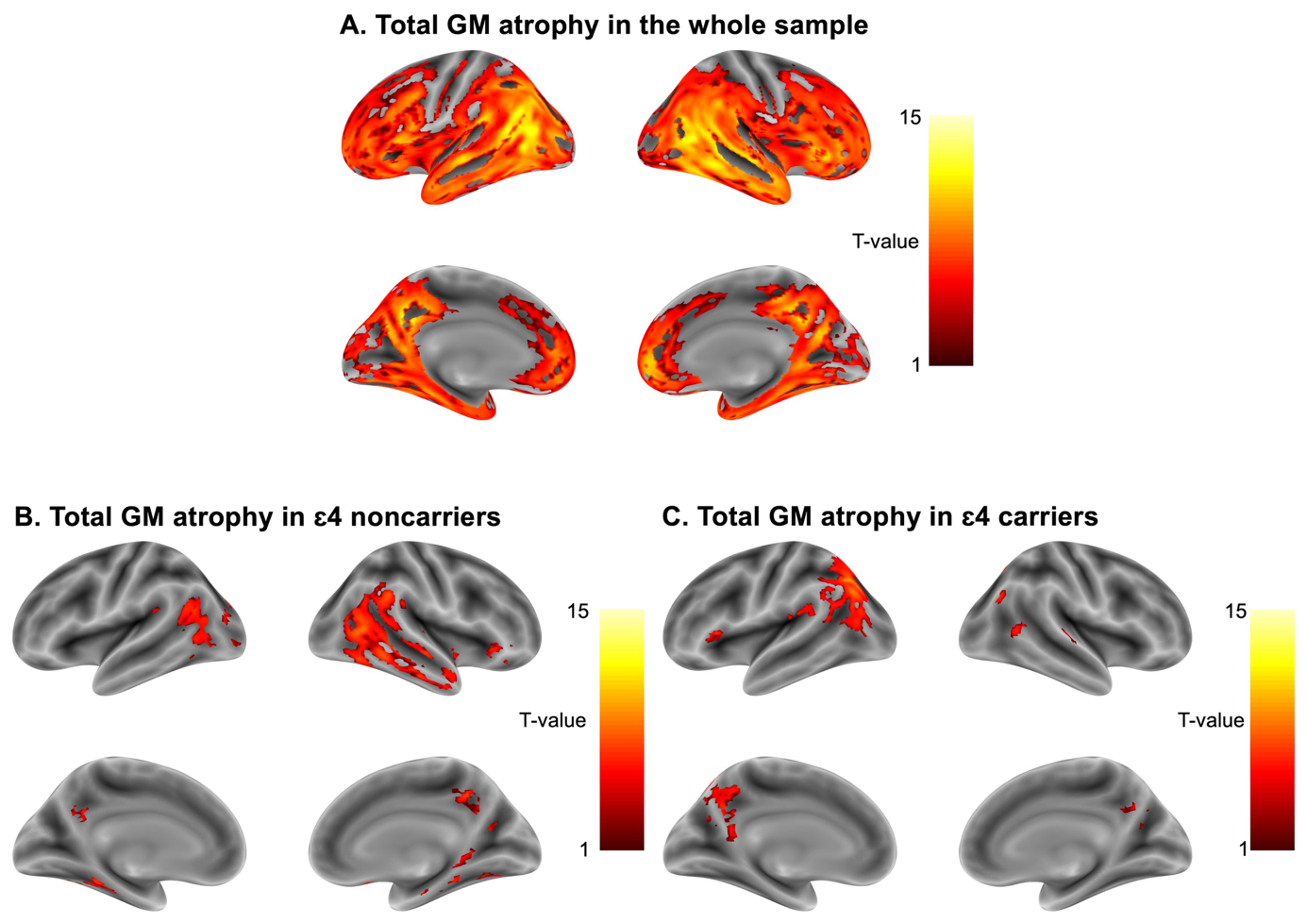


**Supplementary Fig. 3** Total gray matter (GM) atrophy. (A) GM atrophy in the whole sample particularly pronounced temporal areas, in the left precuneus, and the left thalamus. B) GM atrophy in ɛ4 noncarriers pronounced in the right hemisphere. C) GM atrophy in ɛ4 carriers pronounced in the left hemisphere. Statistical threshold was set to *p_FWE_* < 0.05 and a k > 100 voxels cluster extent. Color range displays T-values from 1 (red) to 15 (yellow) showing all significant regions


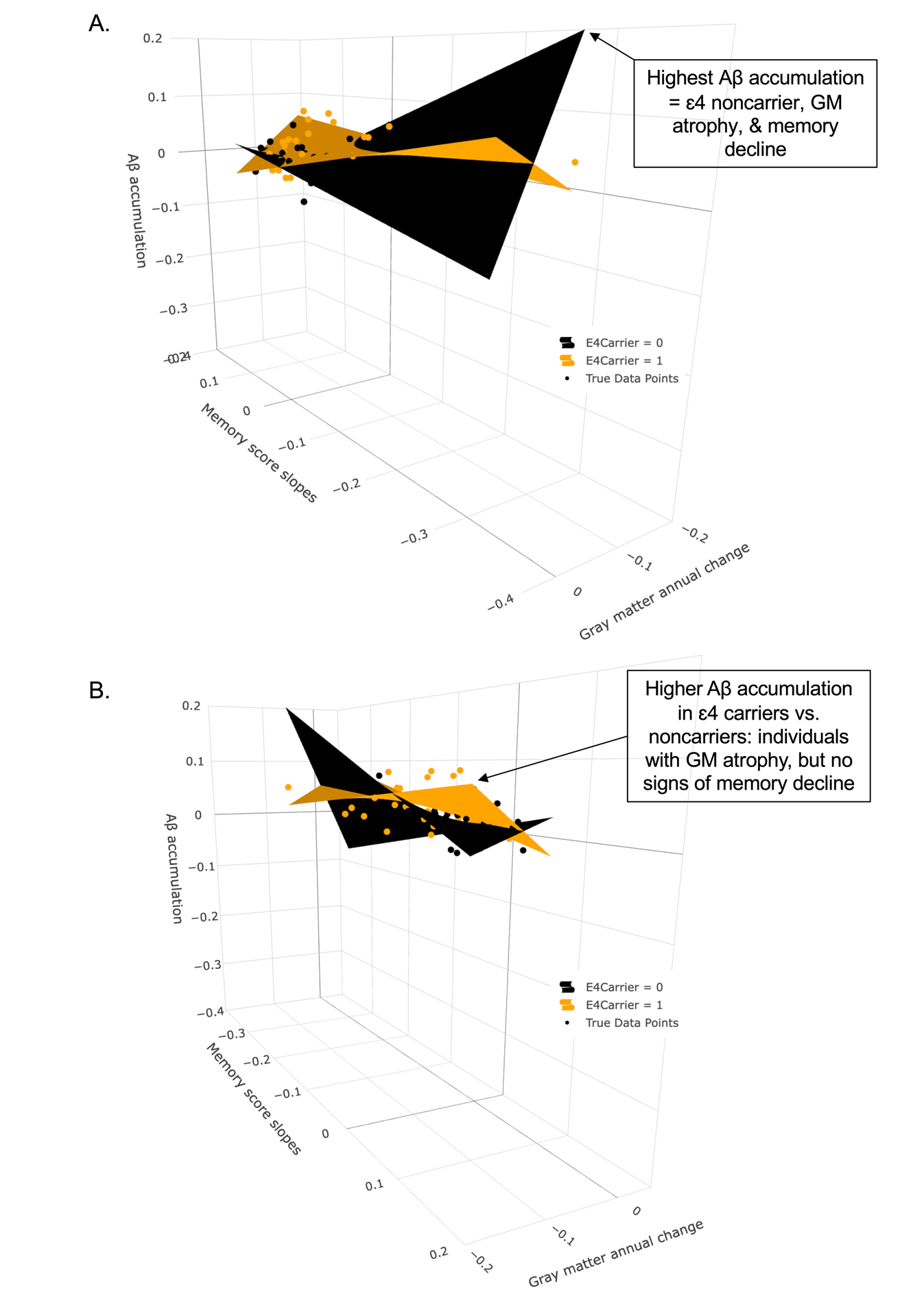


**Supplementary Fig. 4** 3D Visualization of the multimodal association with the APOE genotype and memory decline in the fusiform gyrus**.** Data points represent mean intensities of amyloid beta (Aβ) annual change rates and gray matter (GM) annual change rates extracted from the significant area surrounding the fusiform gyrus and in the occipital cortex. (A) and (B) display different views rotated around the z-axis. The interactive version of this plot can be found [online](https://www.uzh.ch/irem/ssl-dir/Maha/Sup_FIG4_Fusiform_3-way-interaction.html)


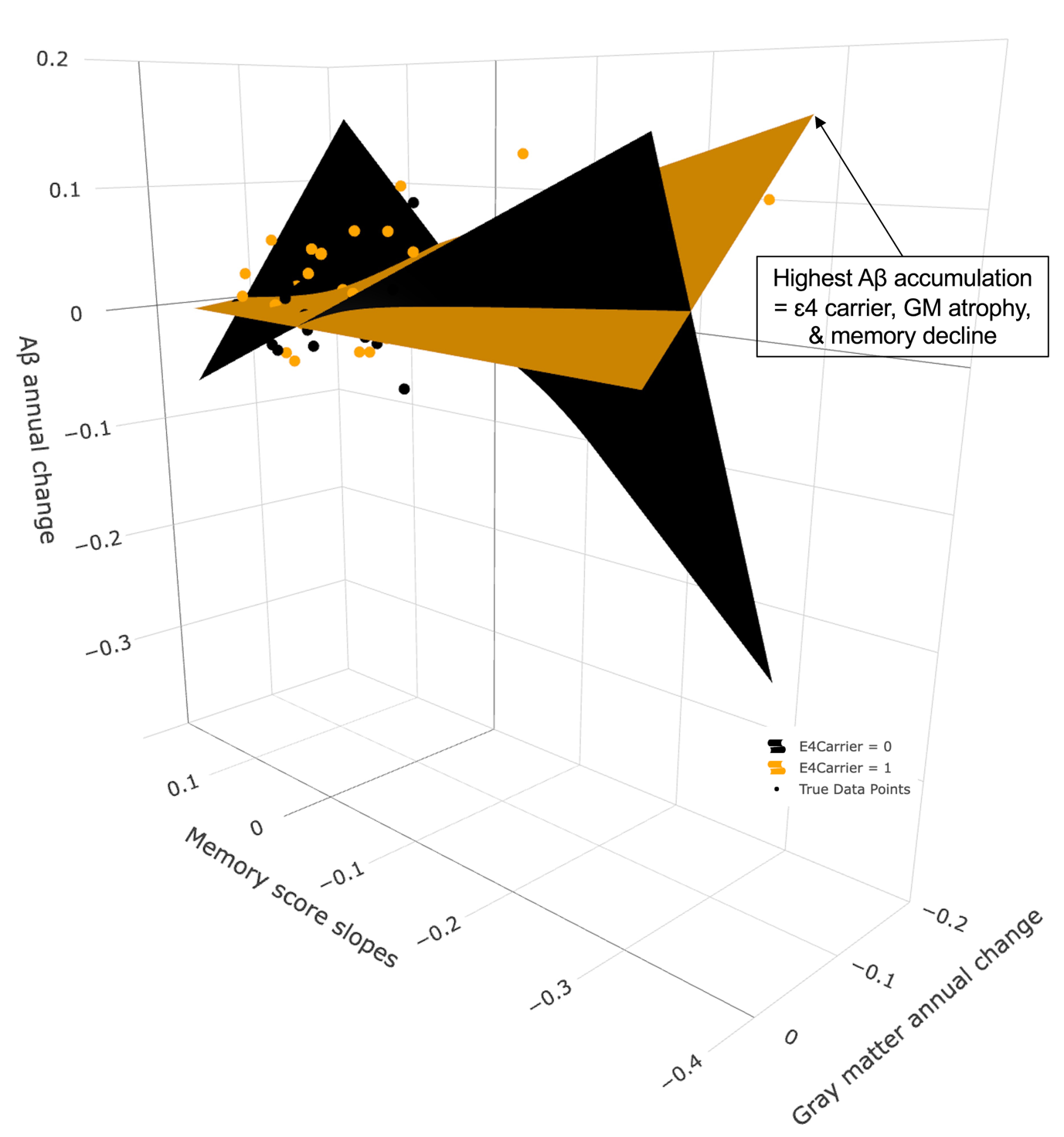


**Supplementary Fig. 5** 3D Visualization of the multimodal association with the APOE genotype and memory decline in the precentral gyrus**.** Data points represent mean intensities of amyloid beta (Aβ) annual change rates and gray matter (GM) annual change rates extracted from the significant area surrounding the precentral gyrus. The interactive version of this plot can be found [online](https://www.uzh.ch/irem/ssl-dir/Maha/Sup_FIG5_Precentral_3-way-interaction.html)


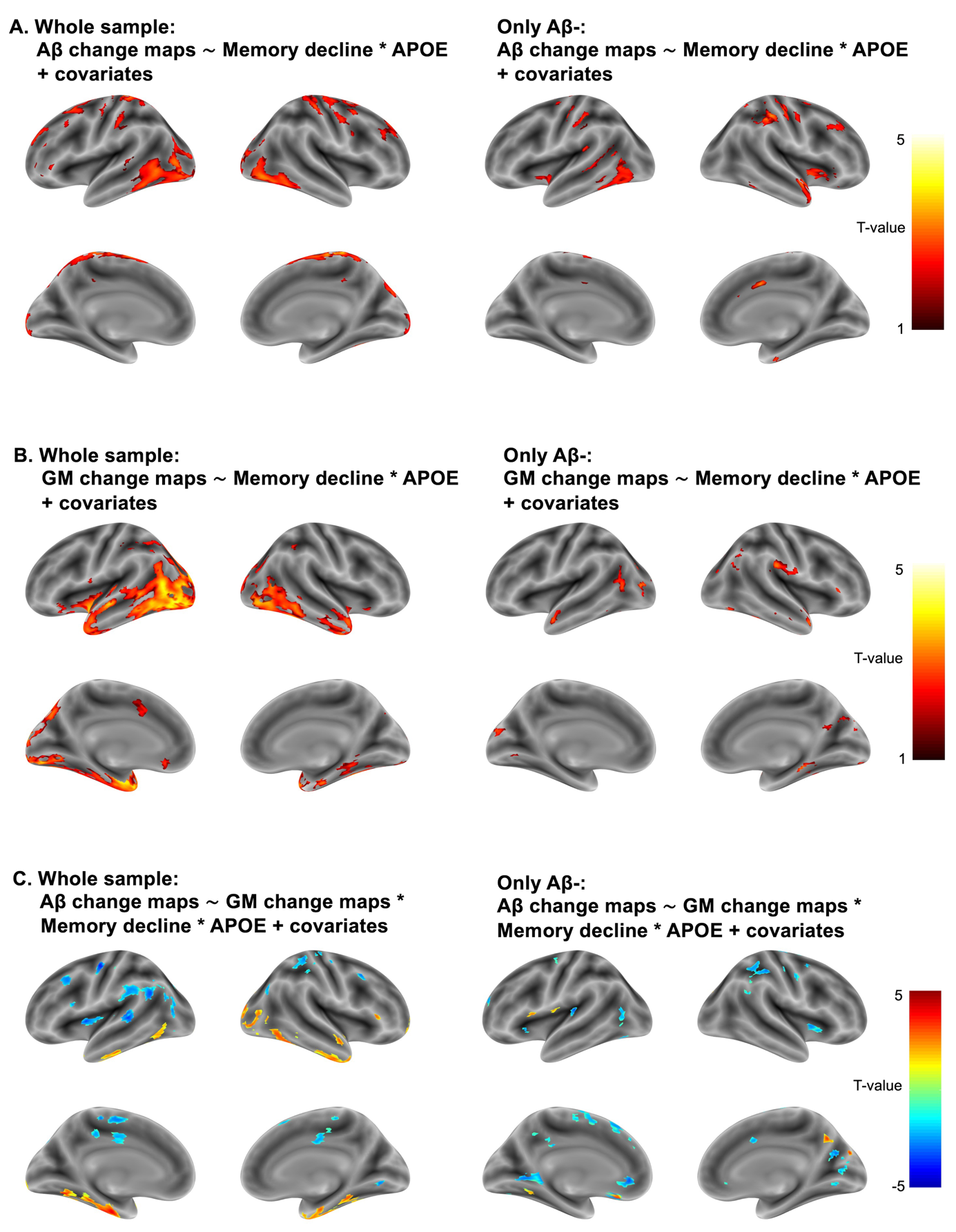


**Supplementary Fig. 6** Voxel-wise analyses in whole sample versus only baseline Aβ normal (Aβ-) subgroup. (A) Interaction of memory decline (binary) and APOE genotype (binary) on Aβ accumulation (change maps). Statistical threshold was set to p_unc_ < 0.001 and a k > 100 voxels cluster extent. Color range displays T-values from 1 (red) to 5 (yellow) showing all regions that were significant at a threshold of p_unc_ < 0.05 and a k > 100 voxels cluster extent. (B) Interaction of memory decline (binary) and APOE genotype (binary) on Gray Matter (GM) atrophy (change maps). Statistical threshold was set to p_unc_ < 0.001 and a k > 100 voxels cluster extent. Color range displays T-values from 1 (red) to 5 (yellow) showing all regions that were significant at a threshold of p_unc_ < 0.05 and a k > 100 voxels cluster extent. C) Multimodal three-way interaction of memory decline (binary), APOE genotype (binary) and GM atrophy (change maps) on Aβ accumulation (change maps). Statistical threshold was set to p_unc_ < 0.001 and a k > 100 voxels cluster extent. Color range displays T-values from -5 (blue) to 5 (red) showing all regions that were significant at a threshold of p_unc_ < 0.05 and a k > 100 voxels cluster extent.
